# Preliminary Characterization of the Epigenetic Modulation in the Human Mesenchymal Stem Cells during Chondrogenic Process

**DOI:** 10.3390/ijms23179870

**Published:** 2022-08-30

**Authors:** Marco Miceli, Giuseppe Maria Maruotti, Laura Sarno, Luigi Carbone, Maurizio Guida, Alessandra Pelagalli

**Affiliations:** 1 Department of Neuroscience, Reproductive and Odontostomatological Sciences, University of Naples “Federico II”, 80131 Naples, Italy; 2 CEINGE Biotecnologie Avanzate, 80145 Naples, Italy; 3 Department of Advanced Biomedical Sciences, University of Naples “Federico II”, 80131 Naples, Italy; 4 Institute of Biostructures and Bioimaging (IBB), National Research Council (CNR), 80131 Naples, Italy

**Keywords:** human mesenchymal stem cell (hMSC), regenerative medicine, histone deacetylase (HDAC) inhibitor, chondrogenic differentiation, epigenetic modulation, cartilage

## Abstract

Regenerative medicine represents a growing hot topic in biomedical sciences, aiming at setting out novel therapeutic strategies to repair or regenerate damaged tissues and organs. For this perspective, human mesenchymal stem cells (hMSCs) play a key role in tissue regeneration, having the potential to differentiate into many cell types, including chondrocytes. Accordingly, in the last few years, researchers have focused on several in vitro strategies to optimize hMSC differentiation protocols, including those relying on epigenetic manipulations that, in turn, lead to the modulation of gene expression patterns. Therefore, in the present study, we investigated the role of the class II histone deacetylase (HDAC) inhibitor, MC1568, in the hMSCs-derived chondrogenesis. The hMSCs we used for this work were the hMSCs obtained from the amniotic fluid, given their greater differentiation capacity. Our preliminary data documented that MC1568 drove both the improvement and acceleration of hMSCs chondrogenic differentiation in vitro, since the differentiation process in MC1568-treated cells took place in about seven days, much less than that normally observed, namely 21 days. Collectively, these preliminary data might shed light on the validity of such a new differentiative protocol, in order to better assess the potential role of the epigenetic modulation in the process of the hypertrophic cartilage formation, which represents the starting point for endochondral ossification.

## 1. Introduction

The loss of organs and tissues, even if partial, due to diseases and traumas, motivates the development of therapies that can regenerate tissues by reducing the dependence on transplants. Regenerative medicine is a branch of translational, interdisciplinary research that applies the principles of tissue and molecular engineering to promote the development of biological substitutes (cells tissue and organs) to restore, maintain or improve normal tissue function or a whole organ [1]. The encouraging preclinical data obtained so far are very promising for the treatment and eradication of both chronic and acute diseases in a wide range of systems and organs [2,3,4]. To achieve these goals, regenerative medicine uses, among others, very innovative techniques such as gene therapy, stem cells, cell reprogramming, and tissue engineering, including the use of new high-tech materials [5].

Mesenchymal stem cells (hMSCs), today more properly called “mesenchymal stromal cells”, are multipotent adult stem cells capable of self-renewal and differentiation into various cell types (neurons, glia, adipocytes, cardiomyocytes, chondrocytes, osteocytes, etc.), both in vitro and in vivo in controlled conditions [6,7,8]. hMSCs can be isolated not only from many adult tissues, including peripheral blood, periosteum, muscles, adipose tissue, connective tissue, skin, bone marrow, and brain, but also from embryonic annexes such as placenta, umbilical cord blood, and amniotic fluid [9,10,11,12].

In addition to this function and to the maintenance of tissue homeostasis in phys-io/pathological conditions, hMSCs are also able to give rise to cells of other tissues (multipotency), even outside the embryonic sheet they belong to (trans-differentiation) [13,14,15,16,17].

Recently, being the hMSC secretome, a principal source of autocrine/paracrine bioactive factors (cytokines, chemokines, growth factors, etc.) [18,19,20,21,22,23], it has been evaluated for its capability to regulate the immune response. This finding result is important to monitor the efficacy in reparative processes (anti-inflammatory and immunomodulatory effects) in various pathological conditions [24,25,26,27].

In this study, hMSCs derived from the amniotic fluid of pregnant women between the 17th and 21st weeks were used, subjected to a diagnostic test of amniocentesis at the request of the gynecologist (prenatal genetic diagnosis), who signed the informed con-sent for the purpose of the research.

In this work, we decided to use hMSCs obtained from amniotic fluid and not cells isolated from many adult tissues, including the bone marrow, mainly because cells found in the amniotic fluid derived directly from the exfoliation of the fetus are considered more “primitive”, maintaining a greater differentiation capacity (see the presence of oct4 and nanog in [9]).

It is well-known that cartilage tissue is a specialized connective tissue, constituted fundamentally by chondrocytes, surrounded by a gelatinous and highly hydrated extracellular matrix (ECM), in which fibrous proteins are immersed; it also allows the diffusion of nutrients, metabolites, and hormones between the blood and the chondrocytes, since the cartilage has no blood vessels. Chondroblasts are housed in gaps in the extracellular matrix where they can divide to form small cell groups (isogenic groups).

The potential for chondrogenic differentiation of hMSCs has been demonstrated in many cell culture systems with or without a matrix [28,29,30,31,32,33,34,35,36,37]. Specifically, in the pellet culture system of Johnstone and Yoo [37,38], hMSCs are “packed” to obtain a high cell density to imitate their behavior during chondrogenesis. These cells are maintained in a defined chondrogenic medium, composed of the transforming growth factor-beta (TGF-β), in the absence of serum. Interestingly, hMSC cultures are subjected to chondrogenesis-expressed genes associated with chondrocyte hypertrophy, including *COL10A1*, alkaline phosphatase (ALP), matrix metallopeptidase 13 (MMP-13), vascular endothelial growth factor (*VEGF*), and parathyroid hormone-related protein receptor (PTHrP-R) [37,38,39,40,41]. This finding suggests that hMSCs chondrogenic differentiation may reach the hypertrophic chondrocytes stage, which normally is characterized by intra-chondral ossification during skeletal development. Chondrocyte hypertrophy in neo-cartilage could ultimately lead to apoptosis, vascular invasion, and ossification, as observed in the cartilage growth plate or epiphyseal plate. Furthermore, after the in vivo ectopic implantation of cartilage constructs derived from hMSCs, the mineralization and vascular invasion or de-differentiation of the chondrocytes takes place, depending on the pre-differentiation status of constructs [41,42,43].

Recently, class II histone deacetylases (HDAC class II) have been reported to act as negative regulators of chondrocyte hypertrophy. It has been hypothesized that HDAC4 could promote TGF-β-induced hMSC chondrogenesis by inhibiting chondrogenically differentiated stem cell hypertrophy. HDAC4’s suppression of chondrocyte hypertrophy differentiates it from class IIa HDACs. Knockout mice lacking HDAC5 or 9 are viable and show no skeletal abnormalities [44,45], while HDAC7-free mice die during average gestation resulting from cardiovascular defects [46]. Instead, HDAC4-null mice show premature ossification of developing bones due to early-onset ectopic chondrocyte hypertrophy, mimicking the phenotype from the constitutive expression of Runx2 in the chondrocytes. In contrast, in vivo HDAC4 overexpression in chondrocyte proliferation inhibits chondrocyte hypertrophy and differentiation, mimicking a Runx2 loss-of-function phenotype [47].

HDACs are the enzymes that catalyze the deacetylation of histone and non-histone proteins and modulate the growth and differentiation of various cell types by governing the structure of chromatin and repressing the activity of specific transcription factors [48,49,50]. The 18 human HDACs are divided into four classes: I (HDAC1, -2, -3, and -8), II (HDAC4, -5, -7, and -9 form the subclass IIa, while HDAC6 and -10 belong to the subclass IIb), III or sirtuins (SIRT1−7), and IV (HDAC11). Classes I, II, and IV HDACs are zinc-dependent enzymes, while class III HDACs are NAD^+^-dependent. Class I HDACs are exclusively nuclear and are believed to act primarily at the chromatin level, while class II HDACs move between the cytoplasm and the nucleus and their expression is tissue-specific [51]. Class I/II/IV HDACs can be recruited from known repressor multiprotein complexes (containing DNA-binding proteins such as Rb and RB-like protein, N-CoR, SMRT, MEF, MeCP2, and sin3A) to suppress transcription of their target genes [52,53,54,55,56,57].

This scenario can be reversed by the use of HDAC inhibitors (HDACi), which activate the transcription of a small set of genes, thus regulating cell proliferation and cell cycle progression [58]. HDACi can be divided into four main classes (hydroxamic acids, cyclic peptides, short fatty acids, and benzamides). Suberoyl anilide hydroxamic acid (*SAHA*), a pan-inhibitor, and *MC1568*, a class II inhibitor of HDAC (selective for HDAC4 and -6), belong to the class of hydroxamic acids, while *MS-275*, an inhibitor of class I, belongs to the benzamides class (Table 1) [51,59,60].

The aim of this preliminary work was to define and establish a new protocol for the differentiation of hMSCs into hypertrophic chondrocytes, which can be used for endochondral or indirect ossification in future bone reconstruction experiments. For this purpose, *MC1568* (belonging to class II deacetylase inhibitors) was used for its selective ability to inhibit HDAC4. *SAHA* and *MS-275* were used as a control of inhibition selectivity.

## 2. Results

### 2.1. Morphological Analysis of the Formation of Compact Three-Dimensional Structures (Spheroid-like) in the Function of the Differentiation Protocols Adopted

Amniocytes were grown in RPMI 1640 at 20% fetal bovine serum (FBS) after a few steps, then split and plated at a high density (1 × 106 cells in 200 µL of the total medium) on a multiple well from 96 to conical bottom. The high cell density and the conical bottom ensured that the cells did not adhere to the plastic of the well but joined together, forming a solid three-dimensional structure, more or less spheroidal, made up of only cells. In the presence of a differentiation medium (DM), adult stem cells were induced towards a chondrogenic fate by specific pathways. This differentiation was further assisted by a high oxygen consumption which led to a situation of strong anoxia, mimicking, as a whole, what occurred in vivo in the cartilage growth plate or epiphyseal plate. The strong anoxia, essential for the differentiation of adult stem cells into chondrocytes, was obtained not only precisely from a very high concentration of cells/mL of the culture medium, but also from the formation of the spheroid, while the cells exposed on the surface of the spheroid were in direct contact with the culture medium, as it descended into the internal layers of the spheroid and the gas exchanges became more and more difficult until it reached the center of the sphere in a total state of anoxia.

Under these conditions, hMSCs, especially those present in the innermost part of the spheroid, changed the morphology and began to synthesize specific components of the extracellular matrix such as glycosaminoglycans (GAGs) [61,62].

For each treatment, the cells were induced with DM for ctr(+), i.e., the standard protocol, or with DM co-induced by HDAC inhibitors (DM + *MS-275*; DM + *SAHA*; DM + *MC1568*), while the ctr(−) cells were grown in RPMI 1640 supplemented with 20% FBS. The medium of each treatment was changed every seven days.

**Figure 1:**
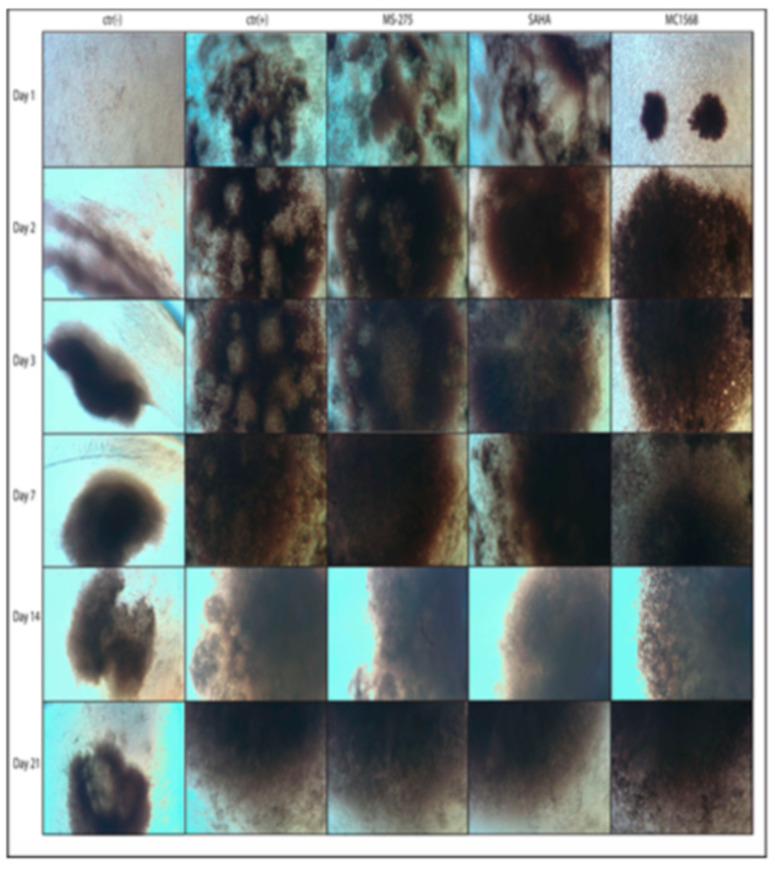
Photos taken 1, 2, 3, 7, 14, and 21 days after the inductions [ctr(−), ctr(+), *MS-275*, *SAHA*, and *MC1568*] of the three-dimensional structures (spheroid-like) that were formed by the condensation of high-concentration hMSCs (1 × 10^6^ cells/200 µL) plated in 96 wells with a conical bottom.

The cells were photographed after 1, 2, 3, 7, 14, and 21 days of induction, to observe the spheroid-like formation in the various treatments. As reported in Figure 1, after one day of plating, the ctr(−) cells were not yet aggregated, while the cells treated with DM or DM plus HDAC inhibitors (*MS-275* or *SAHA*) began to associate, forming clouds of loose cells. At the same time in the group treated with *MC1568*, from the first day, four very compact cell-like spheroids were originated, and they appeared gradually merged into a single larger spheroid-like structure. At 2 days from the plating, the cells of the ctr(−) began to aggregate as well, forming a small very loose cell cloud which gradually thickened more and more as the days went by. At the same time and for the next 21 days, the treated cells formed very dense cell structures and with an increasingly large radius due to the hypertrophy of the cells in the chondro-osteocytic differentiation. It is important to underline that the cells treated with *MC1568* not only were the fastest to aggregate, but also showed a higher cell density especially in the center of the scaffolding that was created, so far as to be clearly visible even with the naked eye.

### 2.2. The Effectiveness of Chondrogenic Stimulation of Amniocytes

Once verified, the ability of the cells to form a compact three-dimensional structure that allowed the development of an anoxic micro-environment such as to favor chondrogenic differentiation, the presence of GAG molecules for the formation of the extracellular matrix typical of chondrocytes was tested.

To demonstrate the presence of GAG in the three-dimensional aggregates, the cells were incubated for three weeks with a specific medium for each of the protocols implemented, namely ctr(−), ctr(+), *MS-275*, *SAHA*, and *MC1568*. The medium of each treatment was changed every seven days.

**Figure 2:**
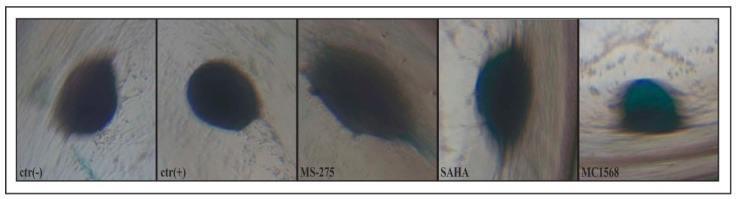
Alcian blue assay performed after 21 days of inductions [ctr(−), ctr(+), *MS-275*, *SAHA*, and *MC1568*] for the detection of the GAGs present in the three-dimensional structures formed by the condensation of the hMSCs.

The three-dimensional structures present after 21 days of incubation were photographed. As shown in Figure 2, all treatments including ctr(−) induced the formation of a three-dimensional structure suitable for differentiation, but only the group of *MC1568* treatment showed a clear dark blue coloring. Differently, in the other wells, including ctr(+), such coloring was not discriminated due to the strong compaction of the cells and the low percentage of GAG fibers present in them.

### 2.3. Analysis of Gene Expression

In order to demonstrate the level of differentiation achieved by the cells during different treatments, from a molecular point of view, the degree of expression of specific markers associated to the endochondral ossification was assessed. In detail, the markers assayed were as follows: *CD44* (marker of chondrogenic differentiation); *COL10a1* (marker of hypertrophic chondrocytes during endochondral ossification); *SPP1*/*osteopontin* (osteoblast marker). Moreover, for a more complete evaluation, the expression levels of both *VEGF* and *bFGF*/*FGF2* were analyzed. The former is a growth factor that induces vascular endothelial cell proliferation and migration, while the latter has mitogenic and angiogenic activity.

A time-course of the 5 markers was carried out at 3, 7, and 14 days in order to check their progresses over time. The medium of each treatment was changed every seven days. For each point of the various treatments [ctr(−), ctr(+), Ms-275, SAHA, and MC1568], five wells were used, in triplicate, for a total of 15 wells (for three independent experiments).

**Figure 3:**
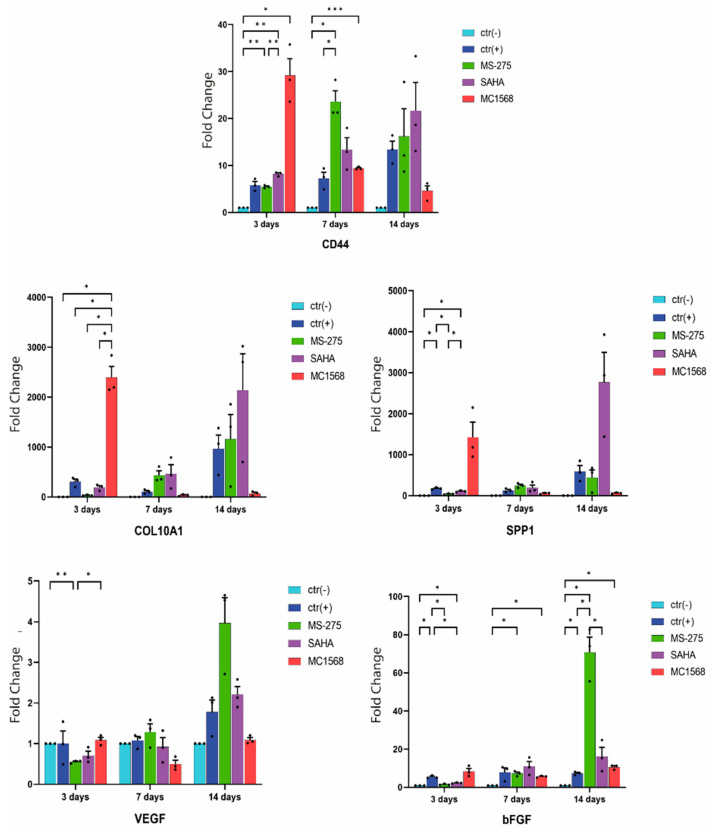
The qRT-PCR analysis for chondrocyte differentiation markers of amniocytes treated or not treated with the different protocols, presented as the fold change (2^−ΔΔCT^) in the level of their expression, which has been normalized to the reference gene GAPDH. The points represent the mean value ± SD (three independent experiments). Two-way ANOVA statistical analyses were performed using GraphPad Prism v9.4.0. The multiple comparison was performed by comparing the mean value of each treated group with the others over time. Tukey’s test was used to correct for multiple comparisons. The asterisks show the significances of the adjusted *p*-values. * *p* < 0.05; ** *p* < 0.01; *** *p* < 0.005.

As shown in Figure 3, CD44 showed a stronger induction already at the 3rd day (relative expression: 28.77 ± 0.493) compared to MC1568, and then, it level fell down on the 7th and the 14th days (relative expressions: 9.40 ± 0.062 and 4.41 ± 0.52, respectively). The other inhibitors and ctr(+) showed an increasing trend over time, but with an up-regulation lower than that already present in MC1568 on the 3rd day. Only MS-275 showed a substantial decrease in relative expression between the 7th and 14th days of induction (from 23.02 ± 0.34 to 14.76 ± 1.05).

A similar trend was observed for COL10a1. In fact, three days after induction, the RNA reached the maximum peak in the cells induced with MC1568 (relative expression of about 2374.38 ± 0.39) and then decreased widely immediately after reaching almost zero between the 3rd and the 14th day of induction. In addition, for COL10a1, the trend of the other inhibitors was uniformly increased. Only ctr(+) demonstrated a slight decrease between the 3rd and 7th days and then returned to grow between the 7th and 14th days.

SPP1 showed a trend similar to that already observed for CD44 and COL10a1. In this case, the cells induced with MC1568 displayed its peak on the 3rd day and then decreased between the 3rd and the 14th days, while both the ctr(+) and the other inhibitors showed an increasing linear trend, with the exception of SAHA which at 14 days showed a strong up-regulation at 14 days of treatment.

As for the expression of *VEGF*, only a small and constant up-regulation occurred in all treatments. Only the induction with *MC1568* showed a different behavior in which between the 3rd and the 7th days there was a down-regulation and then it went up again between the 7th and the 14th days, oscillating between the minimum relative expression of 0.48 ± 0.38 and the maximum relative expression of about 1 ± 0.13.

Finally, in regard to *bFGF*, for all treatments, a moderately increasing up-regulation was observed over time, with only *MS-275* which showed, between the 7th and the 14th days, an up-regulation of about 72 ± 0.1.

## 3. Discussion

The lesions and diseases affecting the bone system are manifold and of different origins: Paget’s disease, osteogenesis imperfecta, osteoarthritis, osteoporosis, bone tumors, trauma, etc., representing one of the most widespread social health problems in the world, causing complex disabilities, significant morbidity, reduced quality of life, severe functional limitations, and death. The management and treatment of these pathologies consequently entails a huge social and economic impact.

Recent scientific progress in cellular and molecular biotechnology has led to the development of therapeutic protocols based on the use of new technologies such as gene therapy, stem cells, cell reprogramming, and tissue engineering, capable of promoting the “process of engineering, regeneration and replacement of human cells, tissues or organs to restore their pre-existing physiological state“ [1]. In particular, experiments on adult stem cells (hMSCs) were very promising.

The formation of bone tissue can occur in two ways: (i) intramembranous or direct ossification; (ii) endochondral or indirect ossification. In both cases, the ossification process involves the replacement of the mesenchymal tissue with bone tissue. In intramembranous ossification, there is a direct passage from the mesenchymal to the bone tissue, while in the endochondral one it passes through an intermediate cartilaginous phase.

The protocols currently in use, for the generation of bone tissue starting from the condensation of adult mesenchymatic cells (direct or indirect ossification), take about 21 days for the differentiation of hMSCs into osteocytes. Unfortunately, this timing is not compatible with most bone generation and replacement interventions after a traumatic injury in which the speed of the intervention can significantly affect the effectiveness of the therapy. Hence, a new and more effective protocol for the development of innovative therapies is needed in the field of regenerative medicine.

The present study aimed to create a new protocol for the regeneration of bone tissue that excludes the use of direct differentiation of hMSCs in osteoblasts and proposed the remodeling of the hypertrophic cartilage template in order to recapitulate the morphogenetic processes of the so-called “endochondral ossification”, typical of embryonic skeletogenesis (indirect ossification), together with the use of HDAC inhibitors with epigenetic effects, which allow the manipulation of the processes underlying the differentiation of hMSCs in hypertrophic chondrocytes in a very short time (less than three days) with a significant reduction in terms of times and costs.

Based on the current knowledge of the main pathways involved in the formation of hypertrophic chondrocytes starting from the condensation of mesenchymatic cells, it has been postulated and then demonstrated that the use of inhibitors of specific classes of HDAC allows modulation, in a targeted way, of some key genes of endochondral ossification.

Members of the TGF-β superfamily play a key role in the different stages of cartilage development. TGF-β up-regulates the gene expression of the transcription factor Sox9, while its down-regulation induces the transition from proliferating chondrocytes to hypertrophic chondrocytes [63], simultaneously inhibiting osteoblast differentiation by repressing Runx2. Recently, class IIa HDACs have been shown to act as powerful negative regulators of chondrocyte hypertrophy; in particular, HDAC4 regulates chondrocyte hypertrophy derived from hMSC condensation [51], interacting and inhibiting Runx2 activity [47]. This repression occurs through the recruitment not only of HDAC4, but also of HDAC5 by the Smad3/Runx2 complex in the Runx2-binding DNA sequence [64]. It has also been shown that HDAC4 knockout mice exhibit premature ossification due to early ectopic hypertrophy. The same result is obtained from the constitutive expression of Runx2 in the chondrocytes. In contrast, HDAC4 overexpression or Runx2 silencing inhibit hypertrophy of chondrocytes in vivo, suggesting the importance of HDAC4 as a central regulator of chondrocyte hypertrophy and skeletogenesis [47].

In this context, it was decided to use some HDAC inhibitors as powerful modulators of gene expression, directing hMSCs already induced by the chondrogenic medium, by the very high cell density towards an osteogenic fate, and by recapitulating the endochondral formation of long bones in vitro.

HDAC inhibitors were chosen based on their specific inhibition characteristics. *MC1568* is a class II HDACi and specifically inhibits HDAC4 and -6 (Table 1), while *MS-275*, a class I HDACi, inhibits HDAC1 and -9 and more weakly HDAC2 and -3 (Table 1). This allowed us to use *MS-275* as a “negative control” of the inhibition compared to *MC1568*. Finally, *SAHA* a powerful pan-inhibitor, in addition to showing a strong inhibition towards HDAC4, strongly inhibited HDAC1, -2, and -3 (class I), HDAC7 and -9 (class IIa), and HDAC6 (class IIb) (Table 1). The inhibition implemented by *SAHA* was considered as an additional “control”, as it was capable to inhibit other HDACs beyond 4.

The data obtained here strongly showed that the specific inhibition of HDAC4 by *MC1568* allowed a faster packing of the hMSCs already at 24 h from induction (formation of the spheroid-like; Figure 1) with a strong anoxia given from the very close cell−cell interactions, while in all the other protocols (ctr(+), *MS-275*, and *SAHA*; Figure 1), the formation of the spheroid-like structure was slower, with cell−cell interaction less present giving their lower compaction and with still high gas exchanges.

Once the higher compaction rate of the cells induced with *MC1568* was determined, we demonstrated that the cells differentiated into pre-hypertrophic (still proliferating) chondrocytes before and hypertrophic chondrocytes after secreting the components of the extracellular matrix such as glycosaminoglycans, thanks to the vital coloring Alcian blue assay (Figure 2). Only in cells induced with *MC1568*, we can see a dark blue coloration due to the greater presence of glycosaminoglycans among the cells, while, in the other wells including ctr(+), this coloring could not be distinguished due to the strong cell compaction coupled to a low percentage of GAG fibers present in them.

Finally, we demonstrated the level of differentiation achieved by the mesenchymatic cells in the different treatments (ctr(−), ctr(+), *MS-275*, *SAHA*, and *MC1568*) from a molecular point of view, photographing the expression level of three important markers at 3, 7, and 14 days of induction, in the three main stages of differentiation, which from proliferating chondrocytes (*CD44*) led to osteocytes (*SPP1*), passing through the stage of hypertrophic chondrocytes (*COL10a1*) (Figure 3). From the data obtained with the qRT-PCR, regarding the cells induced with *MC1568*, it can be seen that in all three markers examined at already three days of induction, the level of expression was at the plateau and then suddenly dropped in the following days. Conversely, in cells induced using other protocols, the expression levels of all three tested markers gradually increased over time, never reaching the expression levels induced by *MC1568*. From these data, it can be deduced that the strong inhibition implemented by the HDACi “*MC1568*” against HDAC4 greatly matched the result obtained by Vega et al. with HDAC4-null mice, in which the lack of HDAC4 produced a phenotype where Runx2 was constitutively expressed in chondrocytes, with the premature formation of developing bones caused by early-onset ectopic chondrocyte hypertrophy [47].

In hMSCs induced with TGF-β3, co-induction with the pan-inhibitor *SAHA*, although presenting, on average, a greater formation of hypertrophic chondrocytes compared to ctr(+), their differentiation occurred in a time comparable to for ctr(+) itself (21 days; Figure 3), demonstrating that the only down-regulation of HDAC4 was necessary and sufficient to accelerate its differentiation towards an osteogenic fate, while the inhibition of other HDACs besides HDAC4 implemented by *SAHA* did not allow it.

Once it was shown that induction with *MC1568* accelerated the formation of hypertrophic chondrocytes, we would like to ascertain whether this induction could also induce the regulation of the main growth factors stimulating angiogenesis and therefore the invasion of blood vessels during endochondral ossification. Both *VEGF* and *bFGF* were found to be unregulated by any of the protocols tested (except for the *MS-275* 21-day induction). This turns out to be a fundamental point of the protocol we designed, as the up regulation of both *VEGF* and *bFGF*, being powerful mitogens, could invalidate its use as a new and more powerful bone regeneration protocol.

The final goal of this project is, therefore, to develop a new and more powerful system for the regeneration of bone tissue that will allow in the near future the “replacement of cells, tissues, organs compromised by diseases, by aging, by congenital or trauma defects” in the shortest possible time.

## 4. Materials and Methods

### 4.1. Amniocytes Cell Culture Conditions and Differentiation Protocol

The amniocytes were obtained from human amniotic fluid-derived hMSCs.

The collection of amniotic fluid or amniocentesis was performed by ultrasound-guided transabdominal puncture between the 17th and 21st weeks of pregnancy. Once withdrawn, amniotic fluid was centrifuged at approximately 1200 rpm for 10 min to concentrate the cells. The supernatant was aspirated from the centrifuge tube, leaving approximately 0.5 mL above the cell pellet (or approximately 2 times the volume of the pellet) of amniotic fluid. The cell pellet was resuspended in a small volume of the patient’s amniotic fluid. Then, about 7 mL of high-content high-glucose RPMI 1640 media (4.5 g/L) (Euroclone, Wetherby, UK), 100 U/mL of pen-strep (Lonza, Verviers, Belgium), 2 mM of L-glutamine (Lonza, Verviers, Belgium) were added, supplemented with 20% fetal bovine serum (FBS) (Euroclone, Wetherby, UK) and kept in an incubator at 37 °C and 5% CO2 in a completely humidified atmosphere.

For chondrogenic differentiation [65], amniocytes cells were cultured in 96-well Clear Round Bottom TC-treated Cell Culture Microplate (Falcon^®^, BD Biosciences, Sunnyvale, CA, USA) at a concentration of 1 × 10^6^ cells/well in 200 µL of a differentiation medium (DM) containing 10 ng/mL of TGF-β3, 100U/mL pen-strep, 2 mM L-glutamine in the absence of a serum in high-glucose RPMI 1640 media (4.5 g/L) for ctr(+) group, 5 µM *SAHA* (MERCK, Readington, NJ, USA) for the “DM + *SAHA*” group, 5 µM *MS-275* (Schering AG, Berlin-Wedding, Germany) for the “DM + *MS-275*” group, and 5 µM *MC1568* (Sigma-Aldrich, St. Louis, MO, USA) for the “DM + *MC1568*” group. Negative control ctr(−) cells were cultured in high-glucose RPMI 1640 media (4.5 g/L) with 20% FBS. The medium of each treatment was changed every 7 days.

The cells were photographed with a 10× magnification optical microscope equipped with a Nikon CoolPix 995 digital camera.

### 4.2. Alcian Blue Assay

Sixty milliliters of ethanol (98–100%) were mixed with 40 mL of acetic acid (98–100%). Ten milligrams of Alcian blue 8 GX (stable solution for 1 year) were dissolved in this solution. One hundred and twenty milliliters of ethanol were mixed with 80 mL of acetic acid (98%–100%) to obtain the bleaching solution.

The process for the assay is shown as follows: the culture medium was gently aspirated from a multiwell taking extreme care not to aspirate the spheroid-like structure. It was washed twice with phosphate buffer saline (PBS) 1× without Ca^++^/Mg^++^. A sufficient amount of neutral buffered formalin (10%) was added to cover the spheroid-like structure, and the mixture was incubated at room temperature for 60 min. Formalin was carefully aspirated and washed 2 times in H_2_O. Distilled water was carefully aspirated, and enough Alcian blue coloring solutions were added to generously cover the cartilage spheroids, as some evaporation occurred. The mixture was incubated overnight at room temperature and in the dark.

The Alcian blue stain solution was carefully aspirated, wash the cells were washed with the bleaching solution for 20 min. The washing step was repeated twice. The bleaching solution was carefully aspirated, and PBS was added.

The cells were then observed and photographed with a 5× magnification optical microscope equipped with a Nikon CoolPix 995 digital camera.

### 4.3. RNA Isolation and Quantitative Real-Time PCR (qRT-PCR)

Total RNA was extracted using TRIZOL reagent (Life Technologies, Monza, Italy) according to the manufacturer’s instructions. RNA concentration and integrity were determined by spectrophotometric measurements using a NanoDrop spectrophotometer (Nanodrop Technologies Inc., Rockland, DE, USA). Then, the RNA (2 µg) was transcribed using the VILO cDNA Synthesis Kit (Invitrogen, Carlsbad, CA, USA) SuperScript according to the manufacturer’s protocol.

Real-Time PCR was performed using iQTM SYBR Green Supermix (Bio-Rad Laboratories Hercules, CA, USA) in a CFX96 Touch Real-Time PCR Detection System (Bio-Rad Laboratories Hercules, CA, USA). The sequences of primers used for cDNA amplification are shown in Table 2. The amplification reactions were performed with 2 µL of cDNA, 0.1 µL of the primer mix (final primer concentration of 0.5 µM each), and 10 µL of 2× iQTM SYBR Green Supermix in a final volume of 20 µL in sterile water. Thermal cycling conditions included an initial phase at 95 °C for 15 min, followed by 40 cycles at 95 °C for 15 s, 62 °C for 30 s, and 72 °C for 30 s. A final dissociation phase was always carried out to obtain the melting curves (thermal profile) of the amplicons obtained in the reactions. All reactions were performed in triplicate, and GAPDH was used as an internal control gene. Gene expression levels were quantified from qRT-PCR data using the comparative threshold cycle (Ct) method. The qRT-PCR analyses for chondrocyte differentiation markers of amniocytes treated or not treated with the different protocols are presented as the fold change (2^−ΔΔCT^) in the level of their expression, which has been normalized to the reference gene GAPDH. The points represent the mean value ± SD (three independent experiments). Two-way ANOVA statistical analyses were performed using GraphPad Prism v9.4.0 [66,67]. The multiple comparison was performed by comparing the mean value of each treated group with the others over time. Tukey’s test was used to correct for multiple comparisons. The asterisks show the significances of the adjusted *p*-values (* *p* < 0.05; ** *p* < 0.01; *** *p* < 0.005).

In Figure 3, only significant comparisons were shown.

## 5. Conclusions and Future Perspectives

One of the fundamental problems for the application of cellular and molecular therapy regarding the regenerative medicine of bone tissue is the differentiation speed of mesenchymal cells, which replaces the diseased or damaged part of the tissue. The protocols currently in use for osteogenic differentiation (direct or indirect) require a time of about 21 days, which represents a very long time considering the impact of speed in bone generation and thus the effectiveness of the therapy.

Our preliminary study proposed a new and more powerful chondro/osteogenic differentiation protocol lasting only three days (compared to 21 days for classic protocols), thanks to the application of “targeted epigenetic modulators” (*MC1568*) that amplify and accelerate formation of osteocytes in a suitable time for the preparation of replacement bone tissue.

The cells thus differentiated were used to “seed” a 3D scaffold in a bio-compatible and bio-degradable material [68] made with a 3D printer [69,70]. This technology can faithfully reproduce the bone morphology to be replaced by the anatomical information obtained from CT or MRI reconstructions.

In the “three-dimensional” structure obtained, the adult stem cells in chondrocyte differentiation occupy, within the scaffold, biological niches created along the bone-like trabeculae which represent, after the surgical implantation, the platform and trigger the formation of a microenvironment in which hypertrophic chondrocytes produce and release specific molecules for the mineralization and vascularization of tissue.

In particular, the metalloproteinases of the matrix (MMP) contribute to the mineralization of the same and the vascular endothelial growth factor (VEGF) to attract blood vessels by distributing osteoblasts, osteoclasts, and hematopoietic pre-cursors, in order to reabsorb the scaffold and promote the formation of bones. These will contain the so-called “stromal sinusoids”, responsible for the formation of the microenvironment suitable for hematopoiesis [71].

At the end of the process described above, both the mesenchymal cells of the scaffold and the scaffold itself will disappear without a trace.

Naturally, since this is a preliminary work, further and more in-depth studies must be conducted for the simultaneous use and in synergy of the most innovative technologies in the field of cell/molecular biology, bioengineering of materials (scaffold), epigenetics, etc. in order to bring the protocol from a purely experimental basis to an effective clinical implementation.

## Figures and Tables

**Table 1:** HDAC inhibitors.

Compound	Inhibition type	HDACi Class	Chemical Structure	Conc. [µM]	Inhibition grade
*SAHA* (vorinostat)	Pan	Hydroxamic Acid	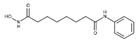	5	High: HDAC1, -2, -3, -4, -6, -7, and -9 Low: HDAC8
*MS-275* (entinostat)	Class I	Benzamides	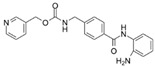	5	High: HDAC1 and -9 Low: HDAC2 and -3
*MC1568*	Class II	Hydroxamic Acid	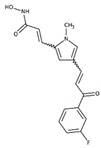	5	High: HDAC4, 6

**Table 2:** Real-time PCR primers.

Primer Name	Primer Sequence
*CD44* Forward	cagggagaaaggggtagtgatac
*CD44* Reverse	tccaagtgagggactacaacag
*COL10a1* Forward	tgcctgtgtctgcttttactg
*COL10a1* Reverse	acccaaacatgagtccctttcac
*SPP1* (*osteopontin*) Forward	tccagtaccctgatgctacag
*SPP1* (*osteopontin*) Reverse	ctctggtcatccagctgactcg
*VEGF* Forward	gagtacatcttcaagccatcctg
*VEGF* Reverse	aggaagctcatctctcctatgtg
*FGF2*/*bFGF* Forward	cagaagagagaggagttgtgtct
*FGF2*/*bFGF* Reverse	ggtgtatttccttgaccggtaag
*hGAPD* Forward	caccatcttccaggagcgag
*hGAPD* Reverse	tcacgccacagtttcccgga

## Abbreviations

ALP	alkaline phosphatase
DM	differentiation medium
ECM	extracellular matrix
FBS	fetal bovine serum
GAG	glycosaminoglycan
hMSC	human mesenchymal stem cells
HDAC	histone deacetylase
MMP-13	matrix metallopeptidase 13
PBS	phosphate buffer saline
PTHrP-R	parathyroid hormone-related protein receptor
*SAHA*	suberoyl anilide hydroxamic acid
SIRT	sirtuin
TGF-β	transforming growth factor-beta
*VEGF*	vascular endothelial growth factor

## References

[1] Mao A.S., Mooney D.J. (2015). Regenerative medicine: Current therapies and future directions. Proc. Nat. Acad. Sci. USA.

[2] Jaklenec A., Stamp A., Deweerd E., Sherwin A., Langer R. (2012). Progress in the tissue engineering and stem cell industry “are we there yet?”. Tissue Eng. Part B Rev..

[3] Bailey A.M., Mendicino M., Au P. (2014). An FDA perspective on preclinical development of cell-based regenerative medicine products. Nat. Biotechnol..

[4] Mendelson A., Frenette P.S. (2014). Hematopoietic stem cell niche maintenance during homeostasis and regeneration. Nat. Med..

[5] Bajaj P., Schweller R.M., Khademhosseini A., West J.L., Bashir R. (2014). 3D biofabrication strategies for tissue engineering and regenerative medicine. Annu. Rev. Biomed. Eng..

[6] Ishikawa F., Shimazu H., Shultz L.D., Fukata M., Nakamura R., Lyons B., Shimoda K., Shimoda S., Kanemaru T., Nakamura K. (2006). Purified human hematopoietic stem cells contribute to the generation of cardiomyocytes through cell fusion. FASEB J..

[7] Lee O.K., Kuo T.K., Chen W.M., Lee K.D., Hsieh S.L., Chen T.H. (2004). Isolation of multipotentmesenchymal stem cells from umbilical cord blood. Blood.

[8] Ohgushi H., Caplan A.I. (1999). Stem cell technology and bioceramics: From cell to gene engineering. J. Biomed. Mater. Res..

[9] Miceli M., Franci G., Dell’Aversana C., Ricciardiello F., Petraglia F., Carissimo A., Perone L., Maruotti G.M., Savarese M., Martinelli P. (2013). MePR: A novel human mesenchymal progenitor model with characteristics of pluripotency. Stem Cells Dev..

[10] Hoogduijn M.J., Crop M.J., Peeters A.M.A., Van Osch G.J.V.M., Balk A.H.M.M., Ijzermans J.N.M., Weimar W., Baan C.C. (2007). Human heart, spleen, and perirenal fat-derived mesenchymal stem cells have immunomodulatory capacities. Stem Cells Dev..

[11] Jordan P.M., Ojeda L.D., Thonhoff J.R., Gao J., Boehning D., Yu Y., Wu P. (2009). Generation of spinal motor neurons from human fetal brain-derived neural stem cells: Role of basic fibroblast growth factor. J. Neurosci. Res..

[12] Krampera M., Marconi S., Pasini A., Galiè M., Rigotti G., Mosna F., Tinelli M., Lovato L., Anghileri E., Andreini A. (2007). Induction of neural-like differentiation in human mesenchymal stem cells derived from bone marrow, fat, spleen and thymus. Bone.

[13] Holden C., Vogel G. (2002). Plasticity: Time for a reappraisal?. Science.

[14] Rice C.M., Scolding N.J. (2004). Adult stem cells-Reprogramming neurological repair?. Lancet.

[15] Okamoto T., Aoyama T., Nakayama T., Nakamata T., Hosaka T., Nishijo K., Nakamura T., Kiyono T., Toguchida J. (2002). Clonal heterogeneity in differentiation potential of immortalized human mesenchymal stem cells. Biochem. Biophys. Res. Commun..

[16] Takeda Y., Mori T., Imabayashi H., Kiyono T., Gojo S., Miyoshi S., Hida N., Ita M., Segawa K., Ogawa S. (2004). Can the life span of human marrow stromal cells be prolonged by bmi-1, E6, E7, and/or telomerase without affecting cardiomyogenic differentiation?. J. Gene Med..

[17] Mori T., Kiyono T., Imabayashi H., Takeda Y., Tsuchiya K., Miyoshi S., Makino H., Matsumoto K., Saito H., Ogawa S. (2005). Combination of hTERT and bmi-1, E6, or E7 induces prolongation of the life span of bone marrow stromal cells from an elderly donor without affecting their neurogenic potential. Mol. Cell Biol..

[18] Salgado A.J., Gimble J.M. (2013). Secretome of mesenchymal stem/stromal cells in regenerative medicine. Biochimie.

[19] Makridakis M., Roubelakis M.G., Vlahou A. (2013). Stem cells: Insights into the secretome. Biochim. Biophys. Acta.

[20] Miceli M., Dell’Aversana C., Russo R., Rega C., Cupelli L., Ruvo M., Altucci L., Chambery A. (2016). Secretome profiling of cytokines and growth factors reveals that neuro-glial differentiation is associated with the down-regulation of Chemokine Ligand 2 (MCP-1/CCL2) in amniotic fluid derived-mesenchymal progenitor cells. Proteomics.

[21] Tran C., Damaser M.S. (2015). Stem cells as drug delivery methods: Application of stem cell secretome for regeneration. Adv. Drug Deliv. Rev..

[22] Skalnikova H., Motlik J., Gadher S.J., Kovarova H. (2011). Mapping of the secretome of primary isolates of mammalian cells, stem cells and derived cell lines. Proteomics.

[23] Pelagalli A., Nardelli A., Lucarelli E., Zannetti A., Brunetti A. (2018). Autocrine signals increase ovine mesenchymal stem cells migration through Aquaporin-1 and CXCR4 overexpression. J. Cell. Physiol..

[24] Abdelrazik H., Giordano E., Barbanti Brodano G., Griffoni C., De Falco E., Pelagalli A. (2019). Substantial Overview on Mesenchymal Stem Cell Biological and Physical Properties as an Opportunity in Translational Medicine. J. Mol. Sci..

[25] Baglio S.R., Pegtel D.M., Baldini N. (2012). Mesenchymal stem cell secreted vesicles provide novel opportunities in (stem) cell-free therapy. Front. Physiol..

[26] Miceli M., Baldi D., Cavaliere C., Soricelli A., Salvatore M., Napoli C. (2019). Peripheral artery disease: The new frontiers of imaging techniques to evaluate the evolution of regenerative medicine. Expert. Rev. Cardiovasc. Ther..

[27] Klimanskaya I., Rosenthal N., Lanza R. (2008). Derive and conquer: Sourcing and differentiating stem cells for therapeutic applications. Nat. Rev. Drug Discov..

[28] Barry F., Boynton R.E., Liu B., Murphy J.M. (2001). Chondrogenic differentiation of mesenchymal stem cells from bone marrow: Differentiation dependent gene expression of matrix components. Exp. Cell Res..

[29] Ichinose S., Tagami M., Muneta T., Sekiya I. (2005). Morphological examination during in vitro cartilage formation by human mesenchymal stem cells. Cell Tissue Res..

[30] Johnstone B., Yoo J.U. (1999). Autologous mesenchymal progenitor cells in articular cartilage repair. Clin. Orthop. Relat. Res..

[31] Li W.J., Tuli R., Okafor C., Derfoul A., Danielson K.G., Hall D.J., Tuan R.S. (2005). A three dimensional nanofibrous scaffold for cartilage tissue engineering using human mesenchymal stem cells. Biomaterials.

[32] Lisignoli G., Cristino S., Piacentini A., Toneguzzi S., Grassi F., Cavallo C., Zini N., Solimando L., Maraldi N.M., Facchini A. (2005). Cellular and molecular events during chondrogenesis of human mesenchymal stromal cells grown in a threedimensional hyaluronan based scaffold. Biomaterials.

[33] Noth U., Tuli R., Osyczka A.M., Danielson K.G., Tuan R.S. (2002). In vitro engineered cartilage constructs produced by press-coating biodegradable polymer with human mesenchymal stem cells. Tissue Eng..

[34] Sekiya I., Vuoristo J.T., Larson B.L., Prockop D.J. (2002). In vitro cartilage formation by human adult stem cells from bone marrow stroma defines the sequence of cellular and molecular events during chondrogenesis. Proc. Natl. Acad. Sci. USA.

[35] Song L., Baksh D., Tuan R.S. (2004). Mesenchymal stem cell-based cartilage tissue engineering: Cells, scaffold and biology. Cytotherapy.

[36] Tuli R., Tuli S., Nandi S., Huang X., Manner P.A., Hozack W.J., Danielson K.G., Hall D.J., Tuan R.S. (2003). Transforming growth factor-betamediated chondrogenesis of human mesenchymal progenitor cells involves N-cadherin and mitogen-activated protein kinase and Wnt signaling cross-talk. J. Biol. Chem..

[37] Yoo J.U., Barthel T.S., Nishimura K., Solchaga L., Caplan A.I., Goldberg V.M., Johnstone B. (1998). The chondrogenic potential of human bone-marrow-derived mesenchymal progenitor cells. J. Bone Joint Surg. Am..

[38] Johnstone B., Hering T.M., Caplan A.I., Goldberg V.M., Yoo J.U. (1998). In vitro chondrogenesis of bone marrow-derived mesenchymal progenitor cells. Exp. Cell Res..

[39] Mwale F., Girard-Lauriault P.L., Wang H.T., Lerouge S., Antoniou J., Wertheimer M.R. (2006). Suppression of genes related to hypertrophy and osteogenesis in committed human mesenchymal stem cells cultured on novel nitrogen-rich plasma polymer coatings. Tissue Eng..

[40] Mwale F., Stachura D., Roughley P., Antoniou J. (2006). Limitations of using aggrecan and type X collagen as markers of chondrogenesis in mesenchymal stem cell differentiation. J. Orthop. Res..

[41] Pelttari K., Winter A., Steck E., Goetzke K., Hennig T., Ochs B.G., Aigner T., Richter W. (2006). Premature induction of hypertrophy during in vitro chondrogenesis of human mesenchymal stem cells correlates with calcification and vascular invasion after ectopic transplantation in SCID mice. Arthritis Rheum.

[42] De Bari C., Dell’Accio F., Luyten F.P. (2004). Failure of in vitro-differentiated mesenchymal stem cells from the synovial membrane to form ectopic stable cartilage in vivo. Arthritis Rheum.

[43] Dell’Accio F., De Bari C., Luyten F.P. (2003). Microenvironment and phenotypic stability specify tissue formation by human articular cartilage-derived cells in vivo. Exp. Cell Res..

[44] Chang S., McKinsey T.A., Zhang C.L., Richardson J.A., Hill J.A., Olson E.N. (2004). Histone deacetylases 5 and 9 govern responsiveness of the heart to a subset of stress signals and play redundant roles in heart development. Mol. Cell Biol..

[45] Zhang C.L., McKinsey T.A., Chang S., Antos C.L., Hill J.A., Olson E.N. (2002). Class II histone deacetylases act as signal-responsive repressors of cardiac hypertrophy. Cell.

[46] Chang S., Young B.D., Li S., Qi X., Richardson J.A., Olson E.N. (2006). Histone deacetylase 7 maintains vascular integrity by repressing matrix metalloproteinase 10. Cell.

[47] Vega R.B., Matsuda K., Oh J., Barbosa A.C., Yang X., Meadows E., McAnally J., Pomajzl C., Shelton J.M., Richardson J.A. (2004). Histone deacetylase 4 controls chondrocyte hypertrophy during skeletogenesis. Cell.

[48] McKinsey T.A., Zhang C.L., Olson E.N. (2002). Signaling chromatin to make muscle. Curr. Opin. Cell Biol..

[49] Thiagalingam S., Cheng K.H., Lee H.J., Mineva N., Thiagalingam A., Ponte J.F. (2003). Histone deacetylases: Unique players in shaping the epigenetic histone code. Ann. N. Y. Acad. Sci..

[50] Pei M., Chen D., Li J., Wei L. (2009). Histone deacetylase 4 promotes TGF-b1-induced synovium-derived stem cell chondrogenesis but inhibits chondrogenically differentiated stem cell hypertrophy. Differentiation.

[51] Nebbioso A., Manzo F., Miceli M., Conte M., Manente L., Baldi A., De Luca A., Rotili D., Valente S., Mai A. (2009). Selective class II HDAC inhibitors impair myogenesis by modulating the stability and activity of HDAC-MEF2 complexes. EMBO Rep..

[52] Ng H.H., Bird A. (2000). Histone deacetylases: Silencers for hire. Trends Biochem. Sci..

[53] Ferreira R., Magnaghi-Jaulin L., Robin P., Harel-Bellan A., Trouche D. (1998). The three members of the pocket proteins family share the ability to repress E2F activity through recruitment of a histone deacetylase. Proc. Natl. Acad. Sci. USA.

[54] Stiegler P., De Luca A., Bagella L., Giordano A. (1998). The COOH-terminal region of pRb2/p130 binds to histone deacetylase 1 (HDAC1), enhancing transcriptional repression of the E2F-dependent cyclin A promoter. Cancer Res..

[55] Nagy L., Kao H.Y., Chakravarti D., Lin R.J., Hassig C.A., Ayer D.E., Schreiber S.L., Evans R.M. (1997). Nuclear receptor repression mediated by a complex containing SMRT, mSin3A, and histone deacetylase. Cell.

[56] Alland L., Muhle R., Hou H., Potes J., Chin L., Schreiber-Agus N., DePinho R.A. (1997). Role for N-CoR and histone deacetylase in Sin3-mediated transcriptional repression. Nature.

[57] Heinzel T., Lavinsky R.M., Mullen T.M., Soderstrom M., Laherty C.D., Torchia J., Yang W.M., Brard G., Ngo S.D., Davie J.R. (1997). A complex containing N-CoR, mSin3 and histone deacetylase mediates transcriptional repression. Nature.

[58] Mai A., Massa S., Rotili D., Cerbara I., Valente S., Pezzi R., Simeoni S., Ragno R. (2005). Histone deacetylation in epigenetics: An attractive target for anticancer therapy. Med. Res. Rev..

[59] Dokmanovic M., Marks P.A. (2005). Prospects: Histone deacetylase inhibitors. J. Cell Biochem..

[60] Heltweg B., Dequiedt F., Marshall B.L., Brauch C., Yoshida M., Nishino N., Verdin E., Jung M. (2004). Subtype selective substrates for histone deacetylases. J. Med. Chem..

[61] Worster A.A., Brower-Toland B.D., Fortier L.A., Bent S.J., Williams J., Nixon A.J. (2001). Chondrocytic differentiation of mesenchymal stem cells sequentially exposed to transforming growth factor-beta1 in monolayer and insulin-like growth factor-I in a three-dimensional matrix. J. Orthop. Res..

[62] Alves da Silva M.L., Crawford A., Mundy J.M., Correlo V.M., Sol P., Bhattacharya M., Hatton P.V., Reis R.L., Neves N.M. (2010). Chitosan/polyester based scaffolds for cartilage tissue engineering: Assessment of extracellular matrix formation. Acta Biomater..

[63] Okubo Y., Reddi A.H. (2003). Thyroxine downregulates Sox9 and promotes chondrocyte hypertrophy. Biochem. Biophys. Res. Commun..

[64] Kang J.S., Alliston T., Delston R., Derynck R. (2005). Repression of Runx2 function by TGF-beta through recruitment of class II histone deacetylases by Smad3. EMBO J..

[65] Xin X., Hussain M., Mao J.J. (2007). Continuing differentiation of human mesenchymal stem cells and induced chondrogenic and osteogenic lineages in electrospun PLGA nanofiber scaffold. Biomaterials.

[66] Manganelli V., Salvatori I., Costanzo M., Capozzi A., Caissutti D., Caterino M., Valle C., Ferri A., Sorice M., Ruoppolo M. (2021). Overexpression of Neuroglobin Promotes Energy Metabolism and Autophagy Induction in Human Neuroblastoma SH-SY5Y Cells. Cells.

[67] Gonzalez Melo M., Fontana A.O., Viertl D., Allenbach G., Prior J.O., Rotman S., Feichtinger R.G., Mayr J.A., Costanzo M., Caterino M. (2021). A knock-in rat model unravels acute and chronic renal toxicity in glutaric aciduria type I. Mol. Genet. Metab..

[68] Xue R., Qian Y., Li L., Yao G., Yang L., Sun Y. (2017). Polycaprolactone nanofiber scaffold enhances the osteogenic differentiation potency of various human tissue-derived mesenchymal stem cells. Stem Cell Res Ther..

[69] Do A.V., Khorsand B., Geary S.M., Salem A.K. (2015). 3D Printing of Scaffolds for Tissue Regeneration Applications. Adv. Healthc. Mater..

[70] Rosenzweig D.H., Carelli E., Steffen T., Jarzem P., Haglund L. (2015). 3D-Printed ABS and PLA Scaffolds for Cartilage and Nucleus Pulposus Tissue Regeneration. Int. J. Mol. Sci..

[71] Scotti C., Tonnarelli B., Papadimitropoulos A., Scherberich A., Schaeren S., Schauerte A., Lopez-Rios J., Zeller R., Barbero A., Martin I. (2010). Recapitulation of endochondral bone formation using human adult mesenchymal stem cells as a paradigm for developmental engineering. Proc. Natl. Acad. Sci. USA.

